# Reflecting on choices and responsibility in palliative care in the context of social disadvantage

**DOI:** 10.1177/26323524231193037

**Published:** 2023-08-25

**Authors:** Maddy French, Lorraine Hansford, Tess Moeke-Maxwell

**Affiliations:** International Observatory on End of Life Care, Lancaster University, Health Innovation Campus, Sir John Fisher Drive, Lancaster LA1 4YW, UK; Wellcome Centre for Cultures and Environments of Health, University of Exeter, Exeter, UK; Te A-rai Palliative Care and End of Life Research Group, University of Auckland, Auckland, New Zealand

**Keywords:** health disparities, health inequalities, public health palliative care, socioeconomic factors

## Abstract

There is a need to understand how to improve palliative care provision for people impacted by social inequity. Social inequity, such as that related to socioeconomic circumstances, has profound impacts on experiences of death and dying, posing personal and professional challenges for frontline professionals tasked to ensure that everyone receives the best standard of care at the end of their lives. Recent research has highlighted an urgent need to find ways of supporting healthcare professionals to acknowledge and unpack some of the challenges experienced when trying to deliver equitable palliative care. For example, those involved in patient or person-centred activities within health settings often feel comfortable focusing on individual choice and responsibility. This can become ethically problematic when considering that inequities experienced towards the end of life are produced and constrained by socio-structural forces beyond one individual’s control. Ideas and theories originating outside palliative care, including work on structural injustice, cultural safety and capabilities approach, offer an alternative lens through which to consider roles and responsibilities for attending to inequities experienced at the end of life. This paper draws upon these ideas to offer a new way of framing individual responsibility, agency and collective action that may help palliative care professionals to support patients nearing their end of life, and their families, in the context of socioeconomic disadvantage. In this paper, we argue that, ultimately, for action on inequity in palliative care to be effective, it must be coherent with how people understand the production of, and responsibility for, those inequities, something that there is limited understanding of within palliative care.

## Introduction

Social inequity has profound impacts on experiences of death and dying, posing personal and professional challenges for frontline professionals tasked to ensure that everyone receives the best standard of care at the end of their lives. There is an urgent need to understand how to improve palliative care provision for people impacted by social inequity, including that related to social deprivation and poverty,^1^ ethnicity and racism,^2^ LGTBQ+ identities^3,4^ and indigenous identities.^3,5^ The phenomena of structural injustice and inequity, however, are unwieldy, pervasive and complex to respond to. Efforts to address them, or at the very least mitigate their impacts, require insight from multiple perspectives and action at multiple levels. Any discrete action taken by one party – for example the redesign of a palliative care model – is more likely to be effective if it is coherent with other actions, and with an overarching appreciation of the complex social, political and economic structures that underpin differences in how people die.

Inequity in the context of healthcare is commonly defined as differences in health, including access to healthcare, that are systematic, avoidable and unfair.^6^ Equity, therefore, is not an abstract unachievable phenomenon but something produced through real life systems and decisions and can be influenced through practical action. Health inequities can manifest across someone’s lifetime, meaning that by the time people are nearing the end of life they may have already experienced multiple forms of inequity throughout their lives. Additionally, some inequities might arise as a consequence of having a terminal illness, for example financial hardship.^7^ Consequently, addressing or mitigating the impact of inequity at the end of life is likely to benefit from action targeting earlier stages in the life course as well as in the last phase of someone’s life.

While often used interchangeable with ‘equality’, there are distinctions between this term and ‘equity’. Equality is the act of treating everyone equally. There are some contexts where this might be considered appropriate, such as the expectation of having equal rights as other citizens in a court of law. Within healthcare, it would not be appropriate to treat everyone equally; people need to be treated in accordance to their needs. Efforts to deliver equitable healthcare, including palliative care, must account for these differences, rather than aspire to ensure everyone receives the same care.

An issue that has arisen from recent research in the United Kingdom concerns the ethical, professional, and practical challenges experienced by people providing palliative care in the context of socioeconomic disadvantage and how they should respond.^8–11^ Finding a way to support healthcare professionals to acknowledge and unpack some of these challenges and tensions is urgently needed, particularly given the pervasive social inequalities in the United Kingdom, including at the end of life.^1,11–14^ Within and beyond the United Kingdom, this objective is relevant for colonised and ethnically marginalised peoples who are affected by racism and cultural marginalisation within the palliative care context.

The purpose of this paper is to explore these challenges, how some professionals respond to them, and consider how incorporating alternative perspectives into palliative care practices could positively impact the care provided to patients and families nearing their end of life in the context of socioeconomic disadvantage.

## Working in the context of inequity

Professionals providing palliative care in the context of socioeconomic disadvantage must navigate the practical and ethical complexities associated with that work.^8,15^ For some this involves considerable ‘hidden work’, including the intensive resource and emotional work that can be needed to support people with psychological, social and material needs arising from lifelong structural disadvantage. This can include managing relationships with other providers to ensure patients get the wider support they are eligible for or spending more time with patients. There may also be challenges building trusting relationships with people who have a rational and reasonable mistrust of services because of prior experiences.^8,10^ This requires professionals to be skilled and motivated to negotiate a way through complicated situations, where existing systems and structures may be more of a hindrance than a help.

**Figure 1.**
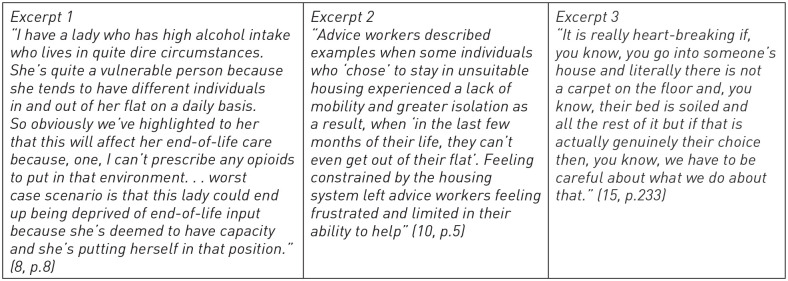
Extracts from research studies.^8,10,15^

Palliative care professionals are often challenged by the constraints of the systems and services they are trying to improve access to. Tensions can arise between the structures and processes associated with a palliative care approach, or a particular model of care, and the preferences, desires or needs of some patients whose lives are different to those of ‘traditional’ recipients of palliative care. Examples of such scenarios, taken from published research conducted in the United Kingdom, are presented in Figure 1. They describe different types of scenarios in which professionals supporting people towards the end-of-life struggle to do so because of a conflict between the lives of people they are supporting and the system, service or professional context in which they are working.

When faced with situations that they felt professionally challenged by, or uncomfortable with, some professionals intuitively focus on the ethics of patient agency, autonomy and individual decision-making. In each excerpt in Figure 1, professionals suggest that someone is choosing to live in circumstances that the professional judges to be unsuitable or problematic for providing palliative or end-of-life care. This includes judgement about the safety or potential misuse of pain medication in a substance-dependent household (Excerpt 1), about a home being unsuitable for care (Excerpt 2), and about the reasons why someone might live in material deprivation (Excerpt 3). As Excerpt 1 and 3 in Figure 1 indicate, if a patient is seen to make a ‘genuine’ choice or to choose to live a certain way, then it can help professionals resolve any tension or uncomfortableness they feel with a situation. This ultimately, and perhaps unintendedly, limits a sense of professional responsibility for a situation considered less than ideal, in one case to the point of someone dying at home in pain with limited professional end-of-life support. This raises questions for the palliative care profession regarding how choice and responsibility are understood and responded to in the context of social inequity and structural disadvantages.^11^ How do palliative care professionals frame choices and decision-making in this context? What is the impact of this framing on patients and families? Are there alternative and better ways of framing situations such as those in Figure 1? This paper aims to move forward with discussion of these areas by exploring the context in which choice narratives have arisen in palliative care and presenting alternative ways of conceptualising them.

## Choice narratives in response to social inequity

That patients have the right to make choices over how they want to be cared for is a central ethic underpinning healthcare policy in many societies, particularly those focused on the rights and responsibilities of individuals. The emphasis on choice in end-of-life care, and across healthcare, corresponds to this larger phenomenon of societies being organised around the idea of maximising individual autonomy. Stemming from a popularisation of the idea of ‘self’ within Western moral philosophy since the Enlightenment, an aspiration for individual autonomy and self-rule became a central characteristic to the dominant liberal thinking in many Western societies, including in bioethics.^16^ With effects of this rippling out into clinical care, the dynamics of clinical interactions shifted to transfer some of the decision-making power from healthcare professionals to patients, challenging the tradition of medical paternalism in the latter half of the 20th century. Consequently, patient autonomy emerged as a dominant ethic in healthcare,^17^ with choice becoming a mechanism by which patients can be empowered to make autonomous decisions including at the end of life.^18^ In past decades, it has subsequently featured in palliative and end-of-life care policies and programmes in several countries, with a focus on helping patients to make decisions about clinical interventions and where they would prefer to be cared for, and to die.^19–25^

Potential problems with narratives around choice and individualism have in recent years been brought to light in the context of growing understanding about the impact of inequities on end-of-life experiences.^1,26^ In cultures where the dominant philosophy is that of individual human rights, agency and individualism, it is important to recognise and respect choices made by any person, even more so for those who have had limited agency throughout their life. However, there is an implicit expectation that self-ruling individuals will not only make choices but will make choices that reflect a particular political and social agenda.^21^ This is extremely problematic when considered in the light of social inequality, which not only impacts the distribution of agency and power^27^ but means that some people are being asked to make choices in line with a political and social agenda that marginalises and excludes them. Palliative and end-of-life care is not immune from this effect. As articulated by Rowley *et al*.^1^ in their paper on poverty at the end-of-life, there is an expectation within palliative care that ‘people will make choices which align with normative understandings of good ways to die’ (p. 7). There is a need, therefore, to ensure that palliative care adapts and responds to the realities of disadvantaged dying, including to situations that challenge professional assumptions of dying well.

## Acknowledging the structural constraints to choices

Patient choice must be understood within the context of social disadvantage. Part of this understanding comes from recognising the social structures and processes that confine the provision of equitable palliative care. Rather than focusing on patient choice, professionals may benefit from focusing on the social forces that patient choices are coming into conflict with. This could be the traditional model or understanding of palliative care but it may also be the social attitudes towards substance use, political apathy towards social housing and care, the reasons Indigenous peoples might be impoverished, or for the historic neglect of care for people with a mental illness. The challenge for palliative care, and any healthcare profession, is to understand this bigger picture and relate it to the situation in front of them, using this to contextualise the choices available and decisions taken by people.

There is a lengthy history of theoretical and empirical work outside the field of palliative care that can be drawn upon to help contextualise individual agency within social structures, and understand their relation to inequity. Others have argued that drawing from highly developed theoretical and academic fields beyond the traditional remit of palliative care, such as the critical poverty literature, can help to reframe the problems faced in practice.^28^ Within the field of health equity, efforts to account for both individual agency and structural factors in the production of inequalities led to the adoption, for example, of the capabilities approach to understanding this phenomenon. The ‘capability’ or ‘capabilities’ approach described fundamental capabilities that a person needs to live a good quality life.^29–31^ This approach has been used widely to try to understand the production and reproduction of health inequalities, and to acknowledge the duality of individual agency and social structure in that production.^32^ The central premise is that social determinants, or social structures, constrain or facilitate whether people are able to be and do something (their capabilities). It is affirmative, inviting states and other actors to put structures in place that allow people to develop needed capabilities.

A capabilities approach may be useful to think about in the context of patient-centred palliative care and patient-led decision making. Instead of expecting individuals to have the same capabilities, it refocuses on the constraints to capabilities. However, some have cautioned that, in practice, it is easy for initiatives focused on the capabilities, assets or resources held by people or communities to end up de-politicising inequalities, as a focus on individual or local practices can neglect the political and economic-related constraints on those capabilities.^26^ Additionally, there is still a judgement required from an individual (e.g. healthcare professionals) as to what sort of constraints influence the different capabilities people have. Aside from economical consideration of the capabilities needed for a good death^33^ there has been little work on the capabilities approach within palliative care, including considering what the relevant capabilities are and their structural constraints. Without this, it is likely that judgement will continue to rely on healthcare professionals drawing on personal, political and philosophical beliefs about their expectations of society and the individual power someone has over their life.

## From understanding to practicing

Palliative care professionals would benefit from acknowledging the structural constraints on patients’ circumstances and decision-making, but understanding of this can be difficult to translate to practice. Many palliative care professionals, particularly those who live or work in the context of social inequity and structural disadvantage, will be aware of the wider social and political forces underpinning inequities in end-of-life circumstances. It would be remiss of the palliative care profession, however, to not learn lessons from other fields of practice where evidence demonstrates the pervasiveness of discourses and narratives that focus on patient’s individual responsibility to make the ‘right’ health decisions.^34–40^ This includes those who are working within the field of health equity,^39^ public health^37^ or those working in primary care in socially deprived contexts.^35,36^ General Practitioners (GPs) working in some of the most deprived areas of Scotland can intuitively fall back on the idea of patients being individually responsible for changing socially determined health outcomes, perhaps reflecting the limitations of their clinical training.^36^

The tendency among professionals to accept the context in which they are working ‘and deal with issues within these preexisting structures’ is understandable (p. 2),^40^ particularly when professionals may feel helpless in addressing broader structural issues, and professional guidance offers little alternative. For example, a study of GP’s working in low-income areas showed that whilst some doctors were aware that a patient’s mental distress was poverty-related, GP’s described the prescription of anti-depressants or talking therapies as one of the only ways in which they felt able to help the patient, as they could not change their socioeconomic circumstances; they also pointed out that this diagnosis of mental distress as an individual psychological problem was the only route recognised by the NICE guidelines.^41^ In her analysis of health equity practitioners’ understanding of working ‘upstream’, McMahon^39^ found what this meant in practice depended on an individual’s pre-existing understanding of their role and perception of the work they should be doing. This may be a reason why health professionals, faced with the difficult task of responding to the abstract and unwieldy phenomenon of ‘health inequalities’ or ‘working upstream’, tend to reduce this to something discrete and actionable.^39^ Writing about oppression, philosopher Mara Marin^42^ argued that the powerlessness people feel when confronted by the impacts of structural injustice can lead to denial of responsibility and an obligation to respond. This makes it difficult to think of actions and solutions beyond the boundaries of existing remits, roles and responsibilities. However, a central premise in Marin’s work, and that in other political philosophers’ writing on injustice such as Young,^43,44^ discussed below, is that there are actions individuals can take against structural injustice. These wider readings of what it means to be responsible for responding to structural injustice and inequity may help those providing palliative care to reflect on equitable responses to the situations they face in the context of structural disadvantage.

## Responsibility for injustice and implications for action

The excerpts in Figure 1 suggest palliative care professionals are uncertain about their role and responsibility to respond to challenges encountered in the context of socioeconomic disadvantage. As those excerpts, and other literature described above, indicate, there is a need to consider in greater depth what is meant by responsibility and how different attitudes towards responsibility have implications for action. To help with this, we can turn to the work and writings of political philosopher Iris Marion Young,^43^ who has had a substantial influence on contemporary thinking about responsibility for structural injustice.^45^ Young, in a book published posthumously and edited by Martha Nussbaum,^43^ argues that social structures constrain individual actions through legal rules, social norms and material infrastructures, with structures continually reproduced through the actions of individuals, each of whom is limited by their particular social position relative to others. From this understanding of social structure, Young posits that responsibility to address injustices arising from social structure should not be focused on blame-worthy individuals but should take the form of collective political responsibility, with individuals defining their action and role within a collective effort by acknowledging their position in relation to a particular injustice.

Rather than suggesting certain individuals have certain duties, Young ^43^ offers four parameters of reasoning to help people identify their responsibility to attend to different structural injustices. Firstly, she suggests that someone should consider what power they hold, which can help them decide what structural injustice(s) to focus on. Secondly, they can consider how they are privileged by current structures, by identifying which injustice(s) they are benefiting from and focusing action on that. Thirdly, they can consider what they have a personal interest in (e.g. are they a victim of structural injustice). Finally, individuals can identify their responsibility for collective ability by identifying which collective(s) they are already part of. A further component of Young’s theory that may be helpful for those navigating responsibility and roles within professional contexts is the recognition of an individual having multiple social positions. A healthcare worker is a professional, a citizen and also possibly a user or future user of the service they are providing. When faced with a structural injustice in a professional context, a healthcare worker can choose to interpret their responsibility from single or multiple social positions, with implications for different actions they may take.

Young’s work has not been without critique, including of the lack of proportionate blame for structural injustice. For example, when discussing Young’s work in the context of fast fashion, Mckeown^45^ argues that while those who buy cheap clothes have some responsibility for the conditions of fast fashion factories in other areas of the world, the companies involved in the employment and production of those clothes are arguably much more to blame. A comparative situation relevant to palliative care could be the proportioning of blame for the exacerbation of chronic illness by poor housing conditions such as mould or damp; here, the landlord is clearly more to blame than a resident or a healthcare worker. Nonetheless, within such a situation, healthcare workers (for example) can still consider what power, interest and collective ability they have to take action.

Part of the argument being proposed here is that healthcare professionals have individual responsibility to be part of a collective action but are not themselves individually responsible for the structural injustice experienced by patients. This should not be confused with a neoliberal argument that individuals should be free agents responsible for their own circumstances and destiny.^46^ However, structural injustice, argued by Young, is produced through the collective actions of individuals. Given that, it makes sense to talk about changes to structures underpinning inequities through the lens of individuals and their collective actions. To return to the example of caring for someone with advanced chronic illness in a mouldy or damp house, instigating actual structural changes may involve changes to legal frameworks (e.g. strengthening tenants’ rights), social norms (e.g. challenging the normalisation of poor housing in specific neighbourhoods) or material infrastructure (e.g. ensuring sufficient affordable housing stock). Healthcare professionals, of course, can only play a small part in influencing such structural change but they nonetheless, as individuals and part of a moderately powerful collective, do have an important role.

Young’s philosophy has been referenced, although not widely, in relation to equity-informed palliative care. Reimer-Kirkham *et al*.^47^ propose that the work of Young and Nussbaum^43^ and other theorists such as Fraser^48^ can provide a framework for equity-oriented palliative care practice, discussing how these theories provided an underpinning framework for a Canadian project about palliative care experiences of structurally vulnerable populations. Writing about the same project a few years later, Stajduhar^49^ called for reflection on power and privilege in palliative care, with clear links between her arguments and the work of Young and others. There are other examples of how such philosophies can be operationalised in palliative care practice, including removing barriers to palliative care for homeless populations^50^ or initiatives to try to overcome legal restrictions for the use of opioids in sub-Saharan Africa.^51^ The compassionate communities’ approach within public health palliative care also stems from a notion of collective responsibility. However, some have cautioned about assigning everyone the same level of responsibility for improving death and dying experiences without considering structural disadvantage and inequity,^9^ echoing the arguments described above regarding the appropriate portioning of blame for injustice.

Such philosophies complement, and are complemented by, arguments for the adoption of cultural safety within healthcare. To paraphrase Curtis *et al*.’s^52^ definition, cultural safety requires healthcare professionals and organisations to be continually critically conscious about how attitudes, assumptions and biases impact care, acknowledging how historical and social dynamics shape power relationships within contemporary healthcare. Culturally safe practices invite a shift from trying to achieve ‘competency’ in an individual patient’s cultural context to understanding healthcare practices in relation to structures and processes of healthcare environments. For example, restrictive healthcare policies may not be supportive of indigenous experiences at the end of life, such as being with kin and completing activities to help prepare the spirit for transition into death.^53^ In such situations, cultural safety is a potentially transformative approach to help reframe barriers as related to healthcare practices, power and assumptions, rather than indigenous culture. As an approach, it shares some similarities with Young’s argument for acknowledging an individual’s relative position of power and responsibility for action within a structured system. More broadly, these arguments indicate there are multiple frameworks or approaches that can be adopted for those interested in delivering equity-oriented palliative care.

## Applying new perspectives to practice

**Figure 2.**
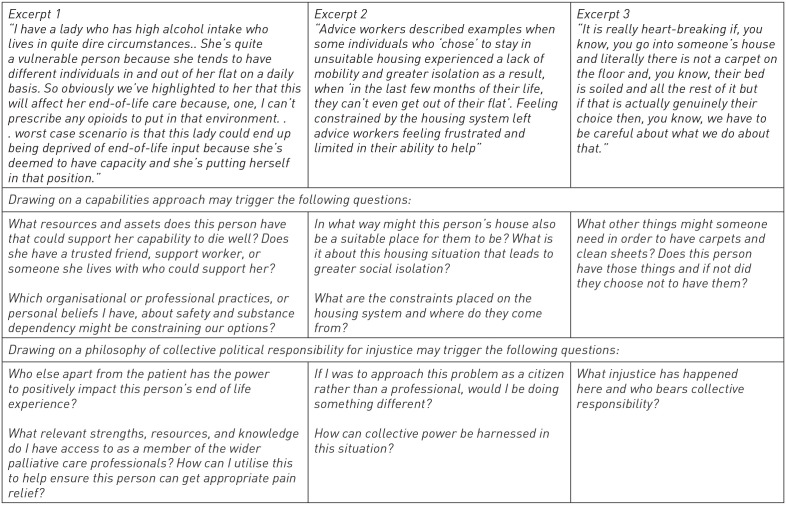
Alternative perspectives on excerpts in Figure 1.

Young’s parameters of reasoning, capabilities theory and cultural safety, are just some approaches that could help orientate palliative care professionals towards new activities and action against structural injustice and disadvantage. What is less clear is how the potentially alternative perspective invited by Young, and which professionals may gain from engaging in the wider philosophies on structural injustice and disadvantage, would change how choice narratives are used in palliative care. Earlier in this paper, extracts from research were presented to demonstrate some of the ways that challenges associated with structural injustice can be framed and responded to by professionals supporting people towards the end of life (Figure 1). These excerpts are presented again in Figure 2 but with corresponding questions to prompt alternative framing and ways of thinking about these situations.

Recognition of the joint role of structure and agency inherent in Young’s work, and more widely in the field of health equity theory,^32^ may help palliative care professionals reframe the tension between patient decision-making and palliative care to one between patient decision-making and structural constraints on choices. However, even with that understanding, palliative care professionals may find themselves in similar positions to those GPs mentioned in studies above, who found it difficult not to focus on patients’ behaviours and decisions when trying to respond to socially determined health outcomes or felt unable to respond beyond a medical remit. Further research and practice-based initiatives are required to understand the links between how inequity is conceptualised in palliative care practice, attitudes towards patients and their social or economic circumstances, and actions taking by professionals.

## Conclusion

There needs to be better understanding of the frameworks, attitudes and philosophies that shape how palliative care professionals respond to social inequities. While this article has focused on palliative care practice, these suggestions may also apply to palliative care research. Researchers themselves may benefit from reflecting on their individual role and responsibility towards collective action on structural injustice, particularly if that is the focus of their research. Examples of research with marginalised or structurally vulnerable groups in Canada, Aotearoa/New Zealand and the United Kingdom show how this might be done responsibly and meaningfully.^54–56^

Focusing on healthcare, there is limited evidence about how palliative care professionals understand structural inequity and its impacts on their actions, initiatives and decisions, although evidence from primary care and public health professions points towards some common discourses. As demonstrated by research from other fields, and emerging within palliative care, those involved in patient or person-centred activities within health settings often feel comfortable focusing on individual responsibility. This can sometimes mean that circumstances or outcomes constrained by socio-structural forces, such as whether someone trusts and accepts input from services, are seen through a more limited framing, as choices and decisions made by individuals.

For action on inequity in palliative care to be effective, however, it must be coherent with how people understand the production of, and responsibility for, those inequities. By drawing on different conceptions of responsibility towards social injustice and structural disadvantage, such as those proposed by Young and other theorists, approaches to patient-led decision-making in palliative care that have a sharper focus on socio-structural constraints can be fostered. Training, education or reflexive practice may help with this and in some settings, cultural safety, structural competency and implicit bias training is already available. Understanding about how to embed ideas such as structural injustice and collective political responsibility into palliative care training remains under-developed, however. How this can be done, and done in a way that leads to changes in practice, needs further thought and examination in both professional practice and research.

## Declarations

**Ethics approval and consent to participate:** Not applicable.

**Consent for publication:** Not applicable.

**Availability of data and material:** Not applicable.
